# Enhancing the Early Differential Diagnosis of Plateau Iris and Pupillary Block Using A-Scan Ultrasonography

**DOI:** 10.1371/journal.pone.0118811

**Published:** 2015-02-17

**Authors:** Yu-Yen Chen, Dachen Chu, Pesus Chou

**Affiliations:** 1 Community Medicine Research Center and Institute of Public Health, National Yang-Ming University, Taipei, Taiwan; 2 Department of Ophthalmology, Yang-Ming University Hospital, Ilan County, Taiwan; 3 Taipei City Hospital, Zhongxing Branch, Taipei, Taiwan; Medical College of Soochow University, CHINA

## Abstract

**Purpose:**

To distinguish the frequently misdiagnosed plateau iris eyes from pupillary block group and normal group, we compared the ocular biometrical parameters of them by A-scan ultrasongraphy.

**Methods:**

In total, we retrospectively reviewed general characteristics and ocular findings including ocular biometric measurements of 71 normal, 39 plateau iris, and 83 pupillary block eyes.

**Results:**

The normal controls, plateau iris group and pupillary block group were significantly different in age, but not in gender. The anterior chamber depth tended to decrease and the lens thickness tended to increase from normal to plateau iris to pupillary block eyes. Compared to those of plateau iris group, the pupillary block group had significantly shallower anterior chamber depth (2.90mm vs. 2.33mm; p<0.001), thicker lens (4.77mm vs. 5.11mm; p<0.001), shorter axial length (23.16mm vs. 22.63mm; p<0.001), smaller relative lens position (2.28 vs. 2.16; p<0.001) and larger lens/axial length factor (2.06 vs. 2.26; p<0.001). However, when comparing plateau iris and normal eyes, only axial length and lens/axial length factor were significantly different (23.16 vs. 23.54; p<0.05 and 2.06 vs. 1.96; p<0.05).

**Conclusions:**

Measured by A-scan ultrasonography, the ocular biometrics of plateau iris were significantly different from those of pupillary block eyes. However, our A-scan ultrasongraphy generally found no significant biometric differences between plateau iris and normal eyes. These findings suggest that while A-scan ultrasonography might be used as a practical tool for differentiating plateau iris and papillary block eyes, a more meticulous gonioscopy and other assessments may be necessary to distinguish plateau iris from normal eyes.

## Introduction

**Fig 1:**
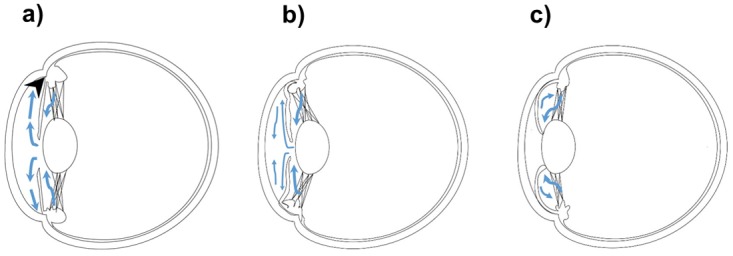
Schematic illustration of the (a) normal eye, (b) plateau iris eye, and (c) pupillary block eye. a) Normal eye. The blue arrow demonstrates the direction of aqueous outflow and the black arrowhead represents the structure of anterior chamber angle. b) Plateau iris eye. It shows convex iris contour and the structure of angle was occluded by the peripheral iris. c) Pupillary block eye. It shows the anterior positioning of the ciliary process and a narrow angle.

Aqueous humor, the water content inside the eye, is produced by the ciliary body, and leaves the eye at the anterior chamber angle (Fig. 1a). If there is angle closure, the outflow pathway will be impeded and the accumulated aqueous inside the eye will cause intraocular pressure to rise. Plateau iris and pupillary block are both classified as angle closure. However, they cause primary angle-closure glaucoma (PACG) through different mechanisms. In frequently misdiagnosed plateau iris, the anteriorly positioned ciliary process pushes the peripheral iris forward, leading to angle crowding [1] (Fig. 1b). In pupillary block, however, the iris-lens contact impedes the aqueous flow from posterior chamber to anterior chamber [2]. The resultant increased pressure gradient between posterior and anterior chamber makes the iris more convex and brings it into apposition with the angle structure [3] (Fig. 1c).

Traditionally, plateau iris and pupillary block were differentiated by gonioscopy and ultrasound biomicroscopy (UBM). Under gonioscopic examination without indentation, both abnormalities have occludable angle. Under indentation gonioscopy, pupillary block eye is recognized by increased iris convexity and peripheral iris apposition to the trabeculum meshwork, and plateau iris has the appearance of double iris hump. When plateau iris is suspected, ultrasound biomicroscopy (UBM) is most often used to collect ocular biometric measurements [4–6]. It shows that the two abnormalities have different morphologies. A plateau iris eye has an anteriorly directed ciliary body, an absent ciliary sulcus, a steep iris root from its point of insertion followed by a downward angulation from the corneoscleral wall, and a flat iris contour. A pupillary-block eye has a convex iris contour with peripheral irido-angle contact under UBM. Plateau iris cannot be treated only as the same way as pupillary block eyes. Iridotomy can relieve the pupillary block component. However, in a plateau iris eye, the angle is still narrow and occludable after iridotomy because iridotomy does not relieve the mechanism of the abnormal ciliary body position.

**Fig 2:**
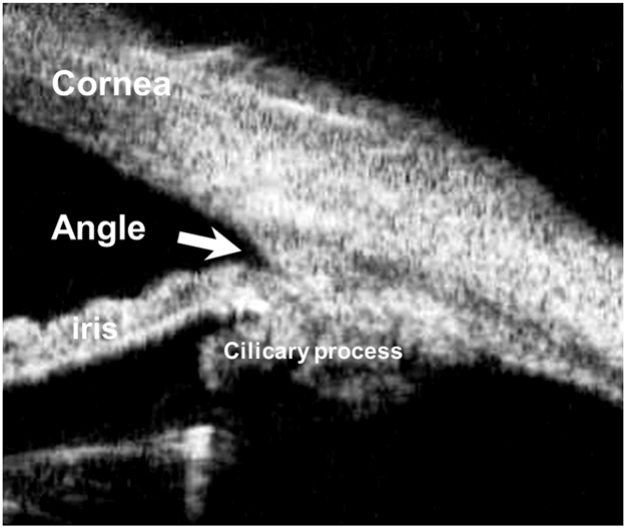
UBM image of an eye. UBM shows the structure of angle. However, it can not demonstrate the structure behind the lens. Thus anterior chamber depth or lens thickness can not be derived.

However, both gonioscopy and UBM are difficult to perform and require experienced ophthalmologists. Relatively, A-scan ultrasonoraphy is more portable and more easily performed. Several studies using A-scan ultrasonography have reported PACG eyes to have a shallow anterior chamber, thickened lens, anterior positioned lens, and shorter axial length [7–10].However, these studies excluded plateau iris eyes, a rarer, more difficult to diagnose abnormality. Some studies used UBM to get information about ocular biometry of plateau iris [4–6].However, UBM could evaluate angle anatomy but could not assess lens thickness or axial length (Fig. 2).

**Fig 3:**
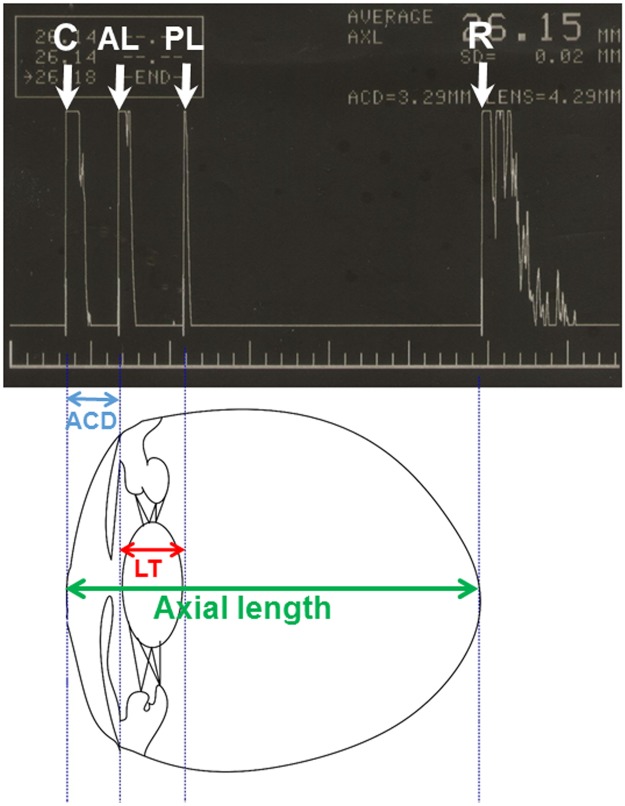
A-scan ultrasonography image along the optical axis of an eye. Peaks correspond to cornea (C), anterior lens (AL) surface, posterior lens (PL) surface, and retina (R). Anterior chamber depth (ACD), lens thickness (LT) and axial length can be derived from A-scan ultrasonography.

A-scan ultrasonography could measure the important structural parameters, such as anterior chamber depth, lens thickness, and axial length (Fig. 3) in normal eyes, plateau iris eyes, and pupillary block eyes. Therefore, in this study, we used A-scan ultrasonography to explore the structural parameters in these eyes. To the best of our knowledge, it is the first study comparing structural parameters in normal eyes, plateau iris eyes, and pupillary block eyes using A-scan ultrasonography. Through the study, we can realize the ocular structural characteristics of these eyes, thus we can further try to differentiate them using A-scan ultrasonography.

## Method

We reviewed the medical charts of patients who visited one doctor at our ophthalmology outpatient department from April 2009 to March 2010. Patients who had been diagnosed with occludable angles by gonioscopy and had pre- and post-iridotomy A-scan data were included as possible study subjects. The diagnosis of plateau iris or pupillary-block eyes was based on the gonioscopy, slit-lamp examination, and ultrasound biomicrosopy (UBM) findings. We also included patients with normal healthy eyes or only with cataract as possible controls. To be included, all patients had to be between 40 and 80 years old and have a refractive error within ± 8D of the spherical equivalent. We excluded all patients who had a history of acute angle-closure glaucoma (AACG) or secondary glaucoma or had received intraocular surgery. We also excluded those who were using miotics, because these drugs might confound the biometric data by moving the lens-iris diaphragm forward. One eye from each participant was selected for analysis using computer-generated random blocks. In total, 193 eyes were enrolled (39 with plateau iris, 83 with pupillary-block, and 71 controls).

We collected general data such as age, gender, body height, body weight, blood pressure, and education level from the medical charts. We also recorded whether the subject had a systemic disease such as diabetes or hypertension.

All participants had medical charts with complete ocular examination data. Ocular examination consisted of visual acuity, refraction, slit lamp examination, fundus examination and intraocular pressure measurement, which was measured by Goldmann tonometer. Angle structure was assessed by goinioscopy using a Goldmann three-mirror goniolens by the same glaucoma specialist, evaluating 360^0^ of the angle of each eye in a dark room. Under gonioscopy without indentation, eyes from the normal group should have open angle, while those from plateau iris and pupillary block groups should have occludable angle, defined as having a pigmented trabecular meshwork that was invisible for more than 180^0^ of its circumference. Under goinscopy with indentation, however, plateau iris eyes would show a sharp peripheral iris drop-off and a double hump sign. Under slit lamp examination, pupillary block eyes would show an iris with a convex contour, which flattened after laser peripheral iridotomy (LPI). A convex iris would not be found in plateau iris eyes. Plateau iris eyes have a flat iris before and after LPI. The diagnosis of plateau iris eyes was confirmed by UBM. Under UBM, plateau iris eyes should show narrow angle associated with anterior positioning of the ciliary processes and the closing of the ciliary sulcus, signs not found in pupillary block eyes.

All patients, including those with normal eyes received A-scan ultrasonograpy during the study period (Digital A/B scan 5500; Sonomed Inc, Lake Success, N.Y., USA). Anterior chamber depth, lens thickness and axial length were measured. Based on these measurement, we further calculated the lens/axial length factor using the formula LT/AL*10. As such, lens/axial length factor could be regarded as a measure of relative lens thickness. In addition, we calculated relative lens position using the formula (ACD+ 0.5*LT)/AL*10. We collected pre-iridotomy A-scan data in the pupillary block patients, but we collected post-iridotomy A-scan data in the plateau iris patients to ensure that data had not been prejudiced by a coexisting pupillary block.

The general characteristics and ocular examination data were summarized descriptively as mean (for continuous variables) and percentages (for categorical variables). The overall differences among the three groups were assessed by one-way analysis of variance (ANOVA). Plateau iris data was compared with the other groups by student’s t test. A p-value less than 0.05 was considered significant. All statistical operations were performed using SPSS version 17.0 (SPSS Inc., Chicago, Illinois, USA). The study was approved by the Kaoshiung Veterens Genetal Hospital IRB committee (2010_08). Due to the retrospective study design, the informed consent was exempt from review according to the IRB, and each patient record was anonymized and de-identified prior to analysis.

## Result

Of the 193 eyes enrolled, 39 were plateau iris, 83 were pupillary block and 71 were normal eyes. As can be seen in Table 1, a summary of general and ocular characteristics of all three groups, patients with plateau iris were younger than those with pupillary-block (mean 61.6 years vs. 65.0 years, respectively). There was no significant difference in male to female ratio was among the three groups (p = 0.62). In addition, there were no significant group differences in body height, body weight and body mass index. Patients in all three groups had similar blood pressure and pulse rate values.

**Table 1:** General and ocular parameters for normal, plateau irs, and pupillary block.

	Normal	Plateau iris	Pupillary-block	p value
	(n = 71)	(n = 39)	(n = 83)	
General characteristics				
Age	64.2±9.8	61.6±9.3	65.0±7.0	p<0.05
Gender				p = 0.62
Male(n, %)	30 (42.3%)	16 (41.0%)	29 (34.9%)	
Female(n, %)	41 (57.7%)	23 (59.0%)	54 (65.1%)	
Education (years)	9.2±5.1	9.5±5.2	7.7±4.5	p<0.05
Body Height(cm)	160.7±8.0	158.9±8.3	157.8±8.6	p = 0.41
Body weight(Kg)	62.4±10.4	60.0±10.4	59.3±10.8	p<0.05
Body mass index	24.1±3.3	23.6±2.9	23.7±3.4	p = 0.08
Diabetes mellitus(n, %)				p = 0.75
No	56 (78.9%)	33 (84.6%)	68 (81.9%)	
Yes	15 (21.1%)	6 (15.4%)	15 (18.1%)	
Hypertension(n, %)				p = 0.25
No	44 (62.0%)	30 (76.9%)	53 (63.9%)	
Yes	27 (38.0%)	9 (23.1%)	30 (36.1%)	
Systolic blood pressure(mmHg)	130.9±17.8	129.1±15.7	133.900B118.8	p = 0.31
Diastolic blood pressure(mmHg)	79.5±13.5	76.4±10.3	79.2±10.6	p = 0.10
Pulse	76.3±10.9	78.5±11.0	77.5±13.4	p = 0.88
Ocular characteristics				
Anterior chamber depth(mm)	2.96±0.21	2.90±0.20	2.33±0.17	p<0.001
Lens thickness(mm)	4.62±0.43	4.77±0.42	5.11±0.35	p<0.001
Axial length(mm)	23.54±0.96	23.16±0.84	22.63±0.80	p<0.001
Relative lens position	2.24±0.14	2.28±0.12	2.16±0.08	p<0.001
Lens/Axial length factor	1.96±0.20	2.06±0.19	2.26±0.16	p<0.001
Cup to disc ratio	0.36±0.09	0.56±0.17	0.66±0.16	p<0.001
Refractive error	0.03±1.99	0.02±2.01	0.36±2.17	p = 0.26
Intraocular pressure(mmHg)	14.3±2.8	15.5±2.9	15.2±3.3	p = 0.16

The three groups had significantly different ocular biometric parameters (all p<0.001). Pupillary block group had the smallest anterior chamber depth, the shortest axial length, as well as the largest lens thickness and lens/axial length factor. The normal eye group fell to the other extreme, and plateau iris group fell in between. The only exception was relative lens position. The pupillary block eyes had the smallest value and the plateau iris group had the largest value. Additionally, the cup-to-disc ratio was significantly different among the three groups (p<0.001), with an increasing trend from normal eyes, plateau iris and pupillary block groups.

We wanted to know what distinguishing differences there might be between plateau iris and pupillary block eyes. As can be seen in Table 2, plateau iris patients were significantly younger (p<0.05). However, both groups were similar in all the other general characteristics. Fundus examination showed pupillary block eyes had a significantly larger cup-to-disc ratio than plateau iris eyes (0.66 vs. 0.56; p<0.01). The two groups also had significant differences in ocular biometric data. Plateau iris eyes had significantly deeper anterior chamber (plateau iris 2.90mm vs. pupillary block 2.33mm), thinner lens (4.77mm vs. 5.11mm), longer axial length (23.16mm vs. 22.63mm), larger relative lens position (2.28 vs. 2.16) and smaller lens/axial length factor (2.06 vs. 2.26). These findings suggest that A-scan sonography can easily distinguish between plateau iris and pupillary block eyes.

**Table 2:** Comparison between plateau iris and pupillary-block.

	Plateau iris vs pupillary block
General characteristics	
Age	p<0.05
Gender	p = 0.52
Education (years)	p = 0.06
Body Height(cm)	p = 0.51
Body weight(Kg)	p = 0.70
Body mass index	p = 0.95
Diabetes mellitus(%)	p = 0.71
Hypertension(%)	p = 0.15
Systolic blood pressure(mmHg)	p = 0.17
Diastolic blood pressure(mmHg)	p = 0.18
Pulse	p = 0.68
Ocular characteristics	
Anterior chamber depth(mm)	p<0.001
Lens thickness(mm)	p<0.001
Axial length(mm)	p<0.01
Relative lens position	p<0.001
Lens/Axial length factor	p<0.001
Cup to disc ratio	p<0.01
Refractive error	p = 0.42
Intraocular pressure(mmHg)	p = 0.65

Because in many of the parameters plateau iris fell between normal eyes and papillary block eyes, we want to find out what difference there might be between plateau iris and normal eyes. As can be seen in Table 3, there was no significant difference in age (p = 0.17) or in any other general characteristic. Plateau iris eyes had shallower anterior chamber, thicker lens, and smaller relative lens position than the normal eyes, though these differences were not significant. The only two significantly different ocular biometric parameters were axial length and lens/axial length factor. Plateau iris eyes had slightly significant shorter axial length (23.16mm vs. 23.54mm; p<0.05) and larger lens/axial length factor (2.06 vs. 1.96; p<0.05). We also compared the measurement of others tests. Plateau iris eyes and normal eyes had similar refraction (p = 0.97), but significantly different cup-to-disc ratio (0.56 vs. 0.36, respectively; p<0.001). Taken together, there were only small differences between plateau iris and normal eyes on A-scan ultrasonograhy.

**Table 3:** Comparison between plateau iris and normal.

	Plateau iris vs normal
General characteristics	
Age	p = 0.17
Gender	p = 0.90
Education (years)	p = 0.75
Body Height(cm)	p = 0.29
Body weight(Kg)	p = 0.26
Body mass index	p = 0.48
Diabetes mellitus(%)	p = 0.46
Hypertension(%)	p = 0.11
Systolic blood pressure(mmHg)	p = 0.60
Diastolic blood pressure(mmHg)	p = 0.21
Pulse	p = 0.32
Ocular characteristics	
Anterior chamber depth(mm)	p = 0.12
Lens thickness(mm)	p = 0.09
Axial length(mm)	p<0.05
Relative lens position	p = 0.17
Lens/Axial length factor	p<0.05
Cup to disc ratio	p<0.001
Refractive error	p = 0.97
Intraocular pressure(mmHg)	p = 0.04

## Discussion

The study compared the general and ocular characteristics among plateau iris, pupillary block and normal eyes. All the general characteristics were similar among the three groups except for age and education. Age increased and education level decreased progressively from plateau iris to control to pupillary block group. The differences in age and in education level were significant between plateau iris and pupillary block group, but not significant between plateau iris and normal group. In all the ocular biometric parameters, plateau iris eyes fell between the normal eye and pupillary block eye groups. While we found significant differences between plateau iris and pupillary block in all of the parameters, the only significant differences between the plateau iris and the normal eye group were axial length and lens/axial length factor.

Plateau iris is less common in Western countries, but it plays a far more important role in PACG in Asia. A cross-sectional study in Singapore evaluated PACG patients over the age of 40 years and found 36 of 111 (30%) PACG eyes in the presence of a patent LPI had plateau iris [11]. Aung T and Kumar RS et al. conducted another study in Singapore which assessed 167 patients of primary angle closure suspects older than 50 years with a patent LPI and found 32.3% of them had plateau iris [12]. A study in China estimated that pure pupillary block only accounts for 38% of angle closure, whereas 54% had combined mechanisms [13]. The Liwan eye study in south China reported that 60% of PACS had a plateau iris with persistent appositional angle closure in the presence of a patent LPI [14]. The studies in Taiwan also reported significant problems of non-pupillary block in hospital-based practice [15,16]. An UBM study in India reported that after LPI in PACG eyes, narrow angles were still persistent in 60% of eyes, of which 67% had an anteriorly positioned ciliary process with a narrow ciliary sulcus [17]. These all highlight the importance of plateau iris of non-pupillary block mechanisms in the Asian PACG eyes. The high prevalence of plateau iris result in the high rate of chronic angle closure and raised IOP even after a successful LPI. Even lens extraction, which is effective to open the angle in pupillary-block eyes, cannot change the iridocilicary apposition in plateau iris patients [18]. In plateau iris eyes, argon laser peripheral iridotomy (ALPI) is the definitive treatment for eliminating residual appositional closure after laser iridotomy [19,20]. If these eyes do not receive proper management, peripheral anterior synechiae will progress due to long-term angle closure and acute/chronic glaucoma will occur, leading to irreversible optic nerve damage. Therefore, it is important to distinguish between plateau iris and pupillary-block eyes as the further treatment differs.

In our study, we found the age of plateau iris group was significantly younger than that of pupillary block group. Previous study conducted by Ritch R. [21] demonstrated that in angle closure patients younger than 40 years old, plateau iris syndrome was the most common etiology (52.2%). Both our study and Ritch’s study highlighted the importance of plateau iris mechanism in angle closure, especially in younger patients. Younger patients has a longer disease course in their future, therefore, early diagnosis in order to receive proper treatment is important. In the past, the diagnosis of plateau iris has been based on gonioscopy with indentation, which shows the double-hump sign. However, it needs a well-trained ophthalmologist to perform the examination and interpret the image correctly. In our study, we tried to find another way to distinguish plateau iris and pupillary-block by comparing the ocular biometry between the two groups. Ocular biometric data were obtained by A-scan ultrasonography, which was portable and easy to perform. We found that each A-scan biometric paremeter was significantly different between plateau iris and pupillary-block. Pupillary-block eyes had smaller anterior chamber depth, axial length, relative lens position and larger lens thickness, lens/axial length factor. Therefore, A-scan biometry might be used as one of the modalities to differentiate plateau iris and pupillary-block.

On the other hand, A-scan biometric data found very few significant differences between plateau iris and normal eyes, suggesting that it might not be a practical tool to distinguish plateau iris from controls. To resolve this problem, however, a slit-lamp evaluation such as Van-Herich test might first be used to distinguish between normal eyes and angle closure glaucoma eyes [22,23]. This could then be followed by A-scan ultrasonogrpahy, which could possibly be used to differentiate between plateau iris and pupilary block eyes. This would reduce the cost and the skill needed to distinguish between the two angle closure glaucoma. Until these preliminary findings are confirmed by further studies, a differential diagnosis should still be confirmed by meticulous evaluation of gonioscopy and UBM.

**Fig 4:**
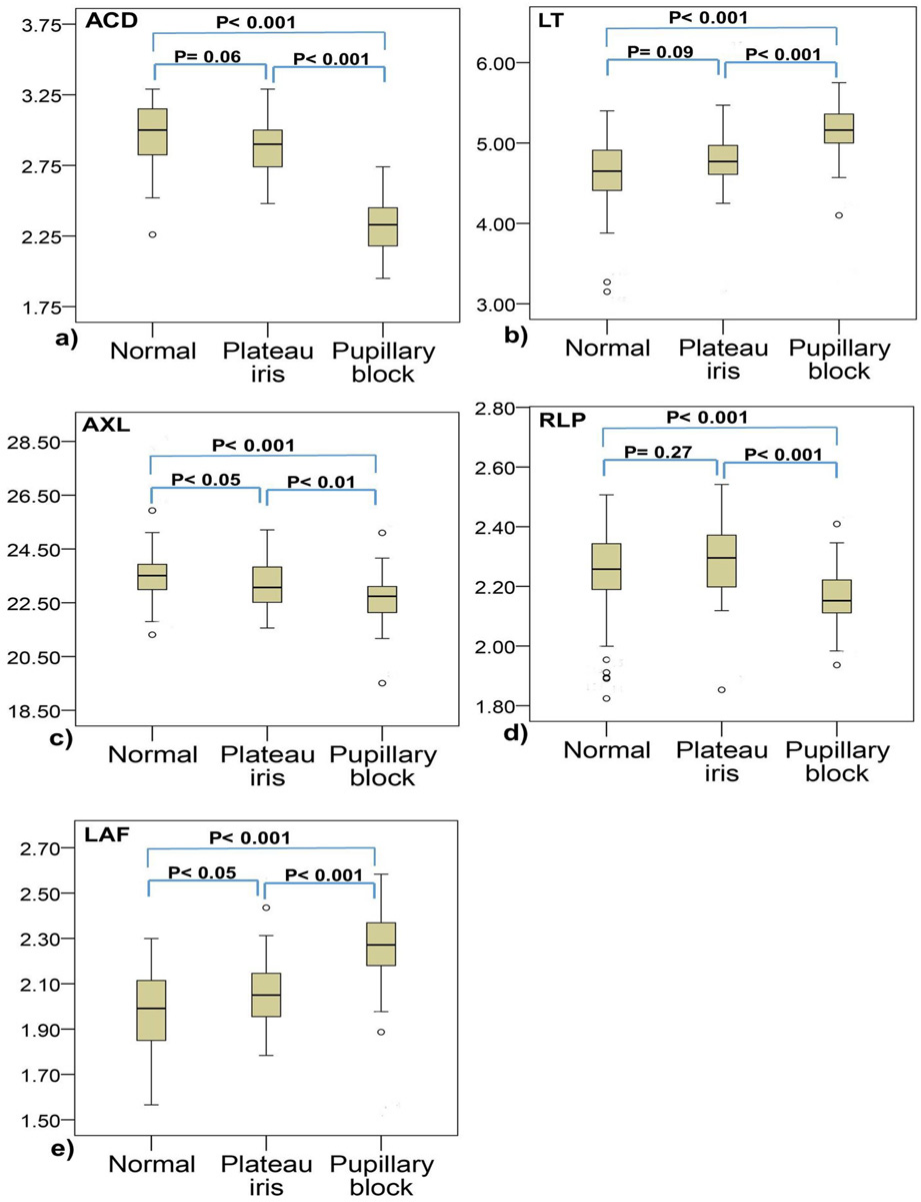
Box-whisker plots of a) ACD, b) LT, c) AXL, d) RLP, and e) LAF. Normal, plateau irs, and pupillary block groups are compared using Kruskal-Wallis test and Wilcoxon rank-sum test. Abbreviations: ACD (anterior chamber depth), LT (lens thickness), AXL (axial length), RLP (relative lens position), LAF (lens/axial length factor).

Our study has some limitations. It was retrospective and data were not collected systematically. The data might be biased because the patients were collected from a tertiary hospital rather than from a population-based study. If a plateau iris eye was not confirmed by UBM examinations, or did not have A-scan data postiridotomy, we exclude the eye. Thus we did not have many case numbers, especially in plateau iris group. It would lead to a lower statistical power, e.g. less than 70% when comparing the A-scan parameters between plateau iris group and normal group in our study. Theoretically, if case number is not enough, there will be a doubt that whether it is statistical enough to draw a reliable result [24,25]. To verify the reliability of our results, we tried to explain it in two perspectives, power analysis perspective and data analysis perspective. In power analysis perspective, we know the definition of power is the probability of correctly rejecting the null hypothesis when it is false. In other words, power means the sensitivity of a statistical test and increasing the sample size will increase the power [26,27]. If one variable is tested to be statistically significant in a study with less sample size, it may become more significant when the sample size increases. And, one variable tested to be statistically non-significant may become significant when sample size increases [28]. Thus, we can have more confidence that in our study, the A-scan ultrasonography parameters tested to be significantly different between plateau iris and pupillary block could be truly different. And, further studies with larger sample size might change the non-significant differences between plateau iris and normal group to be significantly different. In data analysis perspective, we know that a smaller sample size would threat the reasonability of using parametric statistical method such as ANOVA or student’s t test. To solve this problem, we tried to analyze our data using nonparametric statistical method such as Kruskal-Wallis test or Wilcoxon rank-sum test (Fig. 4). We found the results unchanged. It means, the variables which were significant in previous analysis are still significant, and the variables which were non-significant are still not significant. Although statistical power is still less than 80%, the nonparametric method indeed has higher power. Thus we can have an idea that our results are somehow reliable. Further studies with larger sample size are still needed to draw a stronger conclusion. Our study is a pioneer study which provide the ocular biometric characteristics of normal, plateau iris, and pupillary block eyes. It also provides the information about the differential diagnosis of them using A-scan ultrasonograhy.

In conclusion, although A-scan ultrasonography might be used to differentiate plateau iris and pupillary block, it could not distinguish between plateau iris and normal in our study. These findings suggest that while A-scan ultrasonography might be used as a practical tool for differentiating plateau iris and papillary block eyes, a more meticulous gonioscopy and other assessments may be necessary to distinguish plateau iris from normal eyes.
